# Evaluation of Skin Permeation and Retention of Topical Dapsone in Murine Cutaneous Leishmaniasis Lesions

**DOI:** 10.3390/pharmaceutics11110607

**Published:** 2019-11-13

**Authors:** Esther Moreno, Alba Calvo, Juana Schwartz, Iñigo Navarro-Blasco, Elena González-Peñas, Carmen Sanmartín, Juan Manuel Irache, Socorro Espuelas

**Affiliations:** 1 ISTUN Institute of Tropical Health, University of Navarra, Irunlarrea 1, 31008 Pamplona, Spain; emorenoa@unav.es (E.M.); acalvo.5@alumni.unav.es (A.C.); jschwartz@unav.es (J.S.); sanmartin@unav.es (C.S.); 2 Department of Pharmaceutical Technology and Chemistry, University of Navarra, Irunlarrea 1, 31008 Pamplona, Spain; mgpenas@unav.es (E.G.-P.); jmirache@unav.es (J.M.I.); 3 IdisNA, Navarra Institute for Health Research, 31008 Pamplona, Spain; 4 Department of Chemistry, University of Navarra, Irunlarrea 1, 31008 Pamplona, Spain; inavarro@unav.es

**Keywords:** dapsone, topical treatment, cutaneous leishmaniasis, pluronic lecithin emulgel, iron

## Abstract

The oral administration of dapsone (DAP) for the treatment of cutaneous leishmaniasis (CL) is effective, although serious hematological side effects limit its use. In this study, we evaluated this drug for the topical treatment of CL. As efficacy depends on potency and skin penetration, we first determined its antileishmanial activity (IC_50_ = 100 μM) and selectivity index in vitro against *Leishmania major*-infected macrophages. In order to evaluate the skin penetration ex vivo, we compared an O/W cream containing DAP that had been micronized with a pluronic lecithin emulgel, in which the drug was solubilized with diethylene glycol monoethyl ether. For both formulations we obtained similar low flux values that increased when the stratum corneum and the epidermis were removed. In vivo efficacy studies performed on *L. major*-infected BALB/c mice revealed that treatment not only failed to cure the lesions but made their evolution and appearance worse. High plasma drug levels were detected and were concomitant with anemia and iron accumulation in the spleen. This side effect was correlated with a reduction of parasite burden in this organ. Our results evidenced that DAP in these formulations does not have an adequate safety index for use in the topical therapy of CL.

## 1. Introduction

Leishmaniasis is a set of infectious diseases caused by intracellular protozoan parasites of the genus *Leishmania*. Over 12 million people are infected with leishmaniasis and about 1.2 million new cases appear annually [1]. There are three main clinical manifestations depending on the tissues and organs affected by the parasite: cutaneous, mucosal, or visceral leishmaniasis. Focusing on cutaneous leishmaniasis (CL), it causes localized skin lesions that may disappear without treatment or persist, causing severe tissue damage, permanent disfigurement, and serious disability. In general, species that are prevalent in the “old world” (i.e., *Leishmania major* and *Leishmania tropica*) produce limited clinical manifestations compared with “new world” species (i.e., *Leishmania braziliensis*, *Leishmania amazonenesis*, and *Leishmania mexicana*).

Currently, therapy for CL is based on the use of pentavalent antimonials, pentamidine, miltefosine, and amphotericin B (AmB). However, they have variable efficacy, serious side effects, high cost, and prolonged treatment that affects patient adherence. Additionally, parasite strains resistant to some of these treatments have led to failure of therapy in many cases. As these chemical approaches do not guarantee successful treatment of CL, the World Health Organization (WHO) recommends finding more effective oral or topical treatments with relatively less adverse effects [2]. In this context, topical treatments are desirable because of the lower systemic toxicity and costs, the ease of use and accessibility, and the higher patient compliance. Among the topical treatments, paromomycin (PM) has been the drug of choice for more than 50 years. Different formulations have been tested in patients with variable cure rates. A mixture of 15% PM plus 12% methylbenzethonium chloride was used to treat CL caused by *L. braziliensis* and *L. mexicana*, with cure rates around 80% [3]. Furthermore, *L. major* CL patients were treated with 15% PM or PM plus 0.5% gentamicin, with cure rates of 84% and 81%, respectively [4]. In a similar study of patients infected with *Leishmania panamensis*, 67% and a 94% of the lesions were healed after treatment with PM or PM plus gentamicin, respectively. Apart from the differences in efficacy depending on the *Leishmania* species, PM and its combinations also caused erythema, skin irritation, and pain [5]. 

New topical products against CL not only have to exhibit intrinsic antileishmanial activity but also penetrate deeply into the dermis where the infected macrophages are and reside there long enough time to kill *Leishmania* parasites. Additionally, the capability of these products to modulate the immune system towards a bias that favors wound healing without scaring is needed [6]. 

Dapsone (DAP, 4,4-diaminodiphenylsulfone, Table 1) is a sulfone derivative with a dual ability to act as an antimicrobial and anti-inflammatory agent. It was first used in the 1940s to treat leprosy. It was later introduced in the treatment of skin disorders such as acne or dermatitis herpetiformis [7]. Furthermore, its use as an antimalarial and antileishmanial drug has also been described. In 1986, Dogra et al. tried DAP treatment in Indian patients suffering from CL for the first time. A dose of 2 mg/kg administered orally for 21 days produced 80% cure rate, and no relapses were declared after 6 months [8]. In a double-blind study conducted in India, oral DAP was also used successfully in the treatment of CL. Here, 82% of the patients that received 100 mg DAP (approximately 4 mg/kg) twice-daily for 6 weeks were cured at the end of treatment [9]. These results led to oral DAP being recommended as a first-line drug for CL in India. Recently, Indian children with CL lesions due to *L. tropica* were treated with oral DAP at a dose of 20 mg/kg per day for 4–6 weeks, with complete healing in 67% of patients. Moreover, the combination of DAP and rifampicin at the same dose produced a 90% cure rate [10]. Despite its efficacy, its use by oral route is limited by its low water solubility, low bioavailability, and severe toxic effects, including hemolytic anemia and methemoglobinemia [11].

In recent years, topical DAP formulations have appeared as well-tolerated medications with mild side effects. A twice-daily 5% DAP gel demonstrated efficacy and safety in patients with acne vulgaris [12]. Additionally, a twice-daily 5% DAP gel was successfully used to treat papulopustular rosacea [13] and dermatitis herpetiformis [14]. However, topical DAP is approved only for treating acne vulgaris in some countries.

Because oral DAP has shown efficacy in the treatment of CL lesions, and also because of its topical use in inflammatory skin diseases, we hypothesized that topical DAP could have a potential therapeutic profile against CL. For this purpose, in vitro antileishmanial activity and cytotoxicity studies of DAP were carried out. Moreover, studies comparing the capacity of DAP to permeate and penetrate across pig ear skin formulated in a conventional O/W cream or in a pluronic-lecithin-based emulgel (PLE) were performed. Finally, the efficacy of a twice-daily topical DAP treatment in two in vivo models of CL due to *L. major* was determined. To the best of our knowledge, this is the first report evaluating the topical efficacy of this affordable and widely available drug against CL.

## 2. Material and Methods

### 2.1. Materials

Dapsone (DAP), stearic acid, cetylic alcohol, glycerol monoestearate, solid paraffin, and white vaseline were supplied by Fagron (Terrassa, Spain). Liquid paraffin was obtained from Guinama (La Pobla de Valbona, Spain). Lipoid S100^®^ (soybean lecithin) was kindly gifted by Lipoid GMBH (Ludwigshafen, Germany). Amphotericin B (AmB), ethylenediaminetetraacetic acid (EDTA), dimethyl sulphoxide (DMSO), sodium hydroxide, 3-(4,5-dimethylthiazol-2-yl)-2,5-diphenyl-tetrazolium bromide (MTT), methanol, phosphate buffered saline tablets, and Pluronic^®^ F-127 were obtained from Sigma-Aldrich (St Louis, MO, USA). Miglyol 810^®^ and Transcutol^®^ were purchased by Gattefossé (Saint-Priest, France). Aldara^®^ (IMQ 5%) was supplied by 3M Pharmaceuticals (St. Paul, MN, USA). Acetonitrile was provided by Merck (Germany). Water (>18 MΩ/cm resistivity) was obtained from an Ultramatic Type I system (Wasserlab, Spain). All other reagents were of analytical grade and were used without further purification. 

### 2.2. Parasites

*L. major* (clone VI, MHOM/IL/80/Friendlin) and *L. braziliensis* (clone BA788) were maintained at 26 °C and continuously stirred with M199 or Schneider’s modified medium (Sigma, St. Louis, Mo, Canada) supplemented with 10% heat-inactivated fetal bovine serum (FBS) (Gibco, Gaithersburg, MD, USA) and 100 UI/mL of penicillin/streptomycin (Sigma) in flasks. M199 medium was supplemented with 25 mM *N*-2-hydroxyethylpiperazine-*N*’-2-ethanesulfonic acid (HEPES, pH 7.2), 0.1 mM adenine (Sigma), 0.0005% hemin (Sigma), 0.0001% biotin (Sigma), 10% heat-inactivated fetal calf serum (FCS, Gibco), and 100 UI/mL of penicillin/streptomycin. Procyclics were obtained after 1–2 days culture and metacyclics were purified in the case of *L. major* from 5 to 6 days in stationary cultures by treatment with peanut agglutinin (PNA) (Sigma) in order to infect peritoneal macrophages and animals. In contrast, *L. braziliensis* metacyclics were not purified. Briefly, stationary promastigote cultures were washed twice in phosphate buffered saline (PBS, pH 7.4, Gibco), resuspended to 2 mL of simple Roswell Park Memorial Institute (RPMI) 1640 medium (Gibco, Gaithersburg, MD, USA), and incubated with 20 µg/mL of PNA (5 mg/mL in PBS) to purify metacyclics from *L. major*. After 20 min of incubation, 10 mL of RPMI 1640 was added carefully and the suspension was centrifuged (Allegra X-30 centrifuge, Beckman Coulter, Fullerton, CA, USA) at 58× *g* for 5 min. The non-agglutinated promastigotes were collected from supernatants, washed two times in PBS, and used to infect macrophages or animals afterwards. 

### 2.3. Isolation of Mouse Peritoneal Macrophages and Cell Cultures

To obtain mice peritoneal macrophages, BALB/c mice were inoculated with 1 mL of 3% (*w*/*v*) thioglycolate (Sigma). After 3 days, animals were euthanized and 5 mL of cold, simple RPMI 1640 medium were injected into the peritoneal cavity and then recovered with a syringe. Cells were collected from the peritoneal fluid by centrifugation (Allegra X-30 centrifuge) at 524× *g* for 10 min. Then, the pellet was resuspended in RPMI 1640 supplemented with 10% FBS and 100 UI/mL of penicillin/streptomycin and incubated at 37 °C in 5% CO_2_. 

The 3T3 fibroflasts and HaCaT keratinocytes, obtained from the ATCC collection, were cultured in 5% CO_2_ at 37 °C in Dulbecco´s modified Eagle´s medium (DMEM, Gibco) containing 10% FBS, 2 mM l-glutamine (Gibco), and 100 UI/mL of penicillin/streptomycin. 

### 2.4. Animals

The in vivo assays were carried out in female BALB/c mice (Harlan, Spain), weighing approximately 20 g. Animals were kept under conventional conditions with free access to food and water. Animals were housed in groups of five in plastic cages in controlled environmental conditions (12:12 h light/dark cycle and 22 ± 2 °C). This study was conducted according to ethical standards approved (21 October 2014, identification protocol code E15-18(126-14E6)) by the Animal Ethics Committee of the University of Navarra in strict accordance with the European legislation on animal experiments.

### 2.5. In Vitro Activity Against L. major and L. braziliensis Promastigotes

To determine the antileishmanial effect of DAP, MTT assays were carried out. Briefly, 2 × 10^5^ parasites per well were seeded in 96-well plates with different concentrations of DAP. Plates were then incubated at 26 °C for 48 h. After incubation, 20 µL of a MTT solution (5 mg/mL in PBS) was added to each well and plates were incubated at 26 °C for 4 h. Finally, 100 µL of DMSO was added and plates were gently shaken for 30 min to ensure complete solubilization of the formazan crystals. Parasite viability was determined using a microplate reader (iEMS Reader MS, Labsystems, Bradenton, FL, USA) at 570 nm. DAP was dissolved in DMSO and untreated cells were used as control. Wells containing only medium were included to subtract the medium background. Results are expressed as mean ± SD for at least three independent experiments in sextuplicate wells. Half-maximum-effect substance concentration (EC_50_) values were obtained by fitting the data to a dose-effect sigmoid curve using Prism 6.0 software (Graphpad Software Inc., San Diego, CA, USA).

### 2.6. In Vitro Cytotoxicity in Peritoneal Macrophages, Fibroblasts, and Keratinocytes

In vitro cytotoxicity assays were performed in mouse peritoneal macrophages, fibroblasts, and keratinocytes using MTT. Briefly, 2 × 10^5^ peritoneal macrophages, fibroblasts, or keratinocytes were seeded in 96-well plates and incubated at 37 °C for 24 h. Then, different concentrations of DAP were added to wells and plates were again incubated for 48 h. Cells without drug treatment were used as control. After 48 h incubation, 20 µL of MTT (5 mg/mL) was added to wells and plates were incubated for 4 h. Then, 100 µL of DMSO was added after removing the medium from each well and plates were gently shaken for 30 min. Cell viability was determined using a microplate reader (iEMS Reader MS, Labsystems) at 570 nm. Results are expressed as EC_50_ mean ± SD for at least three independent experiments in sextuplicate wells. 

### 2.7. Effect of DAP on L. major and L. braziliensis Amastigotes

Peritoneal macrophages of BALB/c mice were collected as described above. Macrophages in a concentration of 3 × 10^4^ cells/well were seeded in Labtek plates (BD Biosciences, Franklin Lakes, IL, USA) and incubated at 37 °C with humidified atmosphere containing 5% CO_2_. After 24 h incubation, macrophages were washed twice with supplemented RPMI 1640 medium and infected with metacyclic promastigotes of *L. major*, isolated as previously described, at a ratio of 7:1 (parasites/macrophages). For *L. braziliensis* infection, the infection rate was 10:1. Cells were incubated with parasites overnight at 37 °C and 5% CO_2_. Then, wells were washed at least three times with complete RPMI 1640 and treated with different concentrations of DAP (25–1000 µM) for 48 h at 37 °C. AmB at 2 µM was used as positive control of treatment and was previously dissolved in DMSO. After 48 h, wells were washed twice using PBS and then slides were fixed with cold methanol and stained with Giemsa for evaluation (Merck Darmstadt, Germany). The infected macrophage percentage and the number of amastigotes per 100 macrophages were evaluated by counting 200 macrophages using an optical microscope (Nikon Eclipse E400, Y-THM, and Y-THR, Chiyoda-ku, Tokyo, Japan). The experiment was performed three times in duplicate wells and results are presented as EC_50_ mean ± SD. The selectivity index (SI) was calculated as the ratio between cytotoxicity (EC_50_) against peritoneal macrophages and activity (EC_50_) against *Leishmania* amastigotes.

### 2.8. Preparation and Physicochemical Characterization of DAP Formulations

To obtain the O/W DAP cream, 15 g of oily phase containing cetyl alcohol, stearic acid, solid paraffin, liquid paraffin, glyceryl monostearate, and white vaseline (Table 2) were melted in a mortar that was heated in a water bath at 70 °C. Then, water (35 g) was added to the oily phase under agitation with a pistil until the mixture was cooled. Finally, DAP (10% *w*/*w*) was incorporated as a micronized powder. The formulation was stored and protected from light in a plastic container at room temperature. This cream composition was chosen because PM (15% *w*/*v*) solubilized in this vehicle was used as an effective treatment in a CL mice model [15]. 

On the other hand, to prepare the PLE of DAP, lecithin was dissolved in medium chain triglycerides (MCT, Miglyol 810^®^) in an ultrasonic bath and an aqueous solution of Pluronic F-127^®^ was allowed to dissolve overnight at 4 °C. Then, the PLE was prepared on ice by adding the aqueous phase slowly to the oily phase at a volume ratio of 2.9:1.1, respectively (Table 2). Afterwards, DAP (10% *w*/*v*) was dissolved in diethylene glycol monoethyl ether (DEGEE, Transcutol^®^) and immediately incorporated into the emulgel. In this case, the formulation was stored and protected from light in a plastic container at room temperature. 

Viscosity measurements of the cream and PLE were carried out at 32 °C using a Haake Viscotester 550 rotational viscometer with a SV2 rotor (Karlsruhe, Germany) and equipped with a thermostatic bath Thermo Phoenix II (Karlsruhe, Germany). Measures were taken from 0 to 700 1/s. Viscosities at 150 1/s were chosen for comparison and are expressed as Pascal per second (Pa.s). The pH values of the formulations were measured using a digital pH meter (GPL 21, Crison Instruments, Barcelona, Spain). Briefly, 500 mg of the formulations were uniformly dispersed in 50 mL of distilled water, and afterwards pH values were determined. The spreadability tests were carried out using the parallel plate method with modifications [16]. First, 100 mg of the cream and PLE were placed within a circle of 1 cm diameter premarked on a glass plate, over which a second glass plate was placed. A 10 g weight was allowed to rest on the upper glass plate for 5 min. The increase in the diameter due to spreading was noted. All experiments were carried out in triplicate. Organoleptic characteristics of the formulations were recorded in terms of odor, color, texture, consistency, and external appearance. Formulations were also investigated by optical microscopy using a Leica DMD 108 (Leica Microsystems, Wetzlar, Germany). Measurements were performed either directly or at a 1:10 distilled water dilution just after formulation at a 40× magnification.

### 2.9. Stability Studies

The stability assessment of DAP formulations was carried out over a 60 day period of storage at three different temperatures: 4, 25, and 40 °C. On days 15, 30, 45, and 60, pH, spreadability, organoleptic properties, phase separation, drug precipitation, and gravitational stability were evaluated and compared with the initial parameters. For the gravitational stability test, preparations were centrifuged at 25 °C and 2700× *g* for 30 min to assess accelerated deterioration [17].

### 2.10. Preparation of Pig Ear Skin 

Skin tissues were obtained from domestic pig ears in a local slaughterhouse. Ears were taken from the animals and transported to the laboratory within a maximum of 2–3 h. After washing with deionized water, the outer region of the ear was excised with a scalpel and sectioned at a thickness of ca. 1 mm using a dermatome (Aesculap GA 630, Tuttlingen, Germany). Then, skins were stored in Parafilm^®^ (Bemis, Neenah, WI, USA) at −20 °C for no longer than two months. Studies were carried out with intact skin (IS) and damaged skin (without stratum corneum (SC) or epidermis). Before the experiments, the skin was allowed to thaw at room temperature in PBS for 30 min. For damaged skin, a tape stripping procedure was performed. Adhesive tape (Scotch Transparent Tape 600, 3M, St. Paul, MN, USA) was pressed onto the skin and then removed with one quick movement. To determine the number of adhesive applications required to eliminate the SC, 0, 15, 30, and 60 pieces of tape were applied. After that, samples were staining with Mayer’s hemalum (Merck Darmstadt, Germany) and analyzed under a microscope (Olympus BH2, Hamburg, Germany). It was observed that 30 pieces of adhesive tape were necessary to remove all of the SC (taped tripped, TS) [18]. In the studies with dermal membranes (DM, without epidermis), full-thickness skin was immersed in water at 60 °C for 45 s to separate the epidermal layer [18]. 

### 2.11. In Vitro Permeation and Penetration Studies

Permeation studies using Franz diffusion cells were carried out on a MicroettePlusTM apparatus (Hanson Research Corp., Chatsworth, CA, USA). Transcutaneous diffusion was assessed according to OECD guideline 428 [19]. The Franz cell receptor compartments (4 mL) were filled with 30 mM PBS and methanol (70:30) to ensure that sink conditions were maintained over the time course of the experiment. Moreover, the receptor medium was homogenised by magnetic stirring (400 rpm) and maintained at 32 ± 1 °C. Skin biopsies were placed horizontally between the donor and the receptor compartments with the SC, epidermis or dermis side up, ensuring their contact with the receptor medium. The whole device was then fixed with a clamp. All Franz diffusion cell experiments were conducted under infinite doses conditions: 500 mg of the two DAP formulations (cream and PLE) were deposited on 1.74 cm^2^ of skin in the donor compartment. The punch areas were devoid of visible structural changes (scratches, erosion, or scars); as such, skin damage could affect the diffusion and metabolism of the tested compound. The drug diffusion kinetic was evaluated by manual sampling 1 mL aliquots of the receptor fluid at the following predetermined times: 0, 0.5, 1, 2, 3, 4, 6, 8, and 24 h. Each withdrawn aliquot was replaced with an equal volume of receptor phase. Franz cell blank experiments were conducted with the formulations without drugs. At the end of the experiment, the excess formulation was removed and the skin pieces were washed with deionized water and stored at −80 °C until analysis. Skin sections were pretreated with 10 µL of proteinase K (10 mg/mL, Sigma) in 200 µL of Tris-Ethylenediaminetetraacetic acid (TE) buffer for 12 h at 56 °C. Then, the treated samples were vortexed, mixed with 800 µL of acetonitrile, and filtered. Next, 500 µL of the filtrate was evaporated and resolved in 500 µL of mobile phase. The corresponding calibrators were prepared, treating DAP in the same way as skin samples. DAP was quantified by HPLC-UV. Analysis was carried out in a 1200 series LC (Agilent Technologies, Waldbronn, Germany) with a diode-array detector set at 295 nm. The chromatographic system was equipped with a C18 column (150 mm × 4.6 mm, 5 μm particle size; Phenomenex, CA, USA) operating at 40 °C. The mobile phase, pumped at 1 mL/min, was a mixture of methanol and PBS 0.03 M (30:70) in isocratic conditions. For the quantification, calibration curves were made in the following ranges: 0.01–50 µg/mL (for reception medium samples) and 2.5–98 µg/mL for skin samples. All of the calibration curves met the previously established performance criteria (*R*^2^ > 0.999, slopes significantly different from 0 and a relative error (in %) for calibrators <15%). Samples from the receptor compartment were directly injected after appropriate dilution in the mobile phase (PBS/methanol, 70:30). The corresponding standards for calibration were also prepared in the mobile phase. The percentage of DAP recovery from skin samples was 100.2 ± 2.2%, which is largely within the 90–110% range that is considered acceptable. Results are expressed as mean ± SD (*n* = 6 for each skin model), performed for at least two independent experiments. 

The flux of drug permeated (*J*ss, µg/cm^2^·h) was calculated from the slope of the steady-state portion of the permeation profile by linear regression analysis [20]. The lag time (h) was calculated from the back extrapolation of the steady-state portion of the graph. The permeability coefficient (*K*p, cm/h) was also calculated as *J*ss/Cdonor, with Cdonor being the drug concentration applied to the skin surface.

### 2.12. In Vivo Efficacy Studies of DAP Formulations in L. major-infected BALB/c Mice

Animals were infected by subcutaneous inoculation of 10^3^ or 10^5^ infective metacyclic promastigotes of *L. major* in the ear or the base of the tail, respectively. Lesions took different times to reach a similar size, so mice were incorporated into the efficacy assay at different times. After 4–6 weeks, lesions of measurable size (average surface of 10 mm^2^ or volume 5 mm^3^) had developed in mice and topical treatments were initiated. Three different groups (*n* = 8) were evaluated in the tail model of infection: (i) untreated infected mice; (ii) mice treated with DAP cream; and (iii) mice treated with DAP–PLE. Moreover, two groups were evaluated in the ear model (*n* = 5): (i) untreated infected mice; and (ii) mice treated with DAP–PLE. Topical treatments were administered twice daily with 50 mg of DAP cream or DAP–PLE for a period of 30 days. Once treatments were finished, animals were kept for 3 days before sacrifice. Lesions were measured every 3 days with a digital caliper. A paromomycin cream (PMN, 15% *w*/*w*) was used as positive control. Final results were expressed as the mean of the lesion diameter or volume (mm^2^ or mm^3^) ± SD. The parasitic load was quantified by PCR and results are expressed as the median. For this purpose, skin fragments from lesions as well as spleen, liver, and popliteal lymph nodes of infective mice were aseptically removed and conserved at −80 °C until quantification.

### 2.13. Blood Sampling

At treatment points coinciding with blood sampling (days 0, 1, 2, 3, 12, and 18), samples were drawn prior to the morning application of topical DAP. Plasma concentrations of DAP were assayed using HPLC/UV at the same conditions previously described. The mobile phase was a mixture of methanol and PBS 0.03 M at a ratio of 20:80 in isocratic conditions. For the quantification, calibration curves were made in the range of 1–40 µg/mL. The percentage of DAP recovery from blood was 51.2 ± 3.4%. Finally, the determination of hemoglobin and hematocrit was performed in total blood using a ABX pentra 60 hematology system. Results are expressed as mean ± SD (*n* = 6).

### 2.14. Iron Determination in Spleens 

Representative samples of spleen were weighed and later dried in an oven (70 °C) to a constant weight. An accurately weighed sample of dried spleen (100–150 mg) was digested with 6 mL sub-boiling nitric acid (distilled from nitric acid 65%, Merck, Darmstadt, Germany) in a closed acid decomposition microwave system (Ethos Plus, Millestone s.r.l., Sorisole, Italy). Digested samples were then made up to 25 mL with ultrapure deionized water. Iron measurements were performed by flame atomic absorption spectrophotometry (Perkin–Elmer Analyst 800, Norwalk, CT, USA) at 248.3 nm, using a hollow cathode lamp operated at 30 mA and a bandwidth of 0.2 nm. A high-sensitivity nebulizer and an air/acetylene flame with oxidant and fuel flow amounts of 17.0 and 2.0 mL min^−1^, respectively, were used. Digested samples solutions were analyzed in triplicate. Iron detection limit (LOD) was set at three times the standard deviation of the blank reagent and corresponded to 0.11 mg/g (*n* = 6), expressed in terms of dry weight. A recovery study was carried out from the spleen samples to check the accuracy of the analytical method (98.2–103.9%; *n* = 6).

### 2.15. qRT-PCR

Parasite DNA from lesion tissue, spleen, liver, and popliteal lymph nodes was obtained following the Genomic DNA tissue protocol of Nucleospin (NucleoSpin Tissue Macherey Nagel). After DNA obtention, concentrations were measured in a Nanodrop (ND-1000 spectrophotometer, Thermo Scientific, Waltham, MA, USA). One nanogram of DNA was used to quantify *Leishmania* with a qRT-PCR system (Bio-Rad, Hercules, CA, USA) by using iQ SYBR Green supermix (Bio-Rad, Hercules, CA, USA) and primers for minicircle kinetoplastic DNA (kDNA) of *L. major*. The number of kDNA copies was determined by extrapolation from the cycle threshold of each sample on a standard curve of known concentration, which gave the number of parasites × 10^4^. The standard was generated by insertion of the *Leishmania* amplicon in a pCR2.1-TOPO vector (TOPO TA cloning kit; Invitrogen, Carlsbad, CA, USA).

### 2.16. Statistical Analysis

Statistical significance was analyzed using Prism 6.0 software. Differences were tested using the Mann–Whitney test for two groups comparison or the one-way ANOVA with Dunnett´s post hoc test for multiple comparison (in vivo efficacy study), with * *p* < 0.05; ** *p* < 0.01; *** *p* < 0.001.

## 3. Results

### 3.1. In Vitro Antileishmanial Activity and Citotoxicity Studies

**Figure 1:**
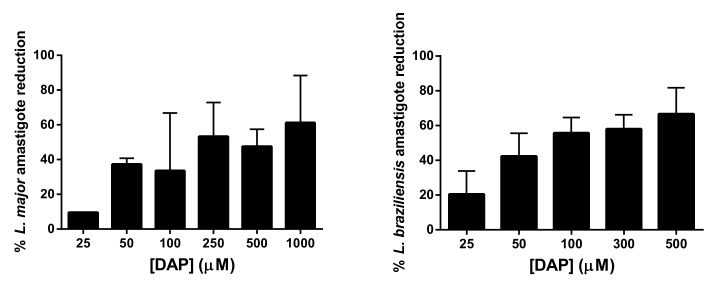
Percentage of *L. major* or *L. braziliensis* amastigote reduction at different concentrations of DAP after 48 h of treatment. Results are expressed as mean ± SD (*n* = 6).

Table 3 shows the antileishmanial activity of DAP. In *L. major* promastigotes, DAP activity was 1.6-fold higher than in *L. braziliensis* promastigotes (EC_50_ at 92 µM vs. 147 µM). Moreover, it showed low toxicity against peritoneal macrophages, fibroflasts, and keratinocytes. We evaluated the cytotoxicity of DAP in these cells, as they are the most frequently found type of skin cells and are also involved in skin repair and wound healing. In fact, the lowest toxicity was found in HaCaT cells (EC_50_ at 3186 µM) followed by peritoneal macrophages (1490 µM) and 3T3 fibroblasts (433 µM) after 48 h of treatment. When DAP was tested against intracellular amastigotes, the activity in *L. major* amastigotes was similar to than in promastigotes (EC_50_ at 92 µM vs. 94 µM). However, in the case of *L. braziliensis*, DAP was 2.7-fold more effective in amastigotes than in promastigotes. Moreover, the number of *L. major* and *L. braziliensis* amastigotes decreased at increasing doses (Figure 1). At 500 µM of DAP, a reduction of 71% was found in *L. braziliensis*, whereas a 45% decrease was obtained for *L. major*. At the lowest dose tested (25 µM), DAP showed 2-fold more activity in *L. braziliensis* than in *L. major* (9.1 µM vs. 20 µM, respectively). Finally, this compound was more selective in killing intracellular parasites than macrophages, showing selectivity index values higher than 15 (Table 3). 

### 3.2. Physicochemical Characterization of the DAP Formulations

The formulations without or containing DAP were characterised in terms of viscosity, spreadability, and pH, and results are presented in Table 4. It can be observed that when DAP was incorporated in the cream, the viscosity and the pH were similar to those in the blank cream (2.46 vs. 2.43 Pa.s and pH 6.52 vs. 6.57). However, the spreadability was slightly decreased (0.85 vs. 0.6 cm). In the case of the PLE formulation, the viscosity was lower when DAP was incorporated in the PLE (3.31 vs. 2.21 Pa.s). In contrast, the spreadability value was 2-fold higher than in the PLE without DAP (1.77 vs. 0.92 cm). Moreover, DAP–PLE showed 3-fold higher spreadability than DAP cream (1.77 vs. 0.6 cm). The spreadability plays an important role in patient compliance and allows uniform application to the skin. According to spreadability data, the PLE DAP formulation was more comfortable and easier to apply over CL diseased skin than the conventional O/W cream. 

Comparing the pH values, the PLE formulations showed lower pH than the cream, but all of them were within the acceptable limits for topical application (between pH 4.8 and 6.6). Concerning the organoleptic characteristics of the DAP cream, it was odorless, had an opaque white color with high consistency, and had no signs of exudation. Moreover, DAP–PLE had a noticeable odor, appeared as opaque light beige, was greasy, soft, and easily spreadable without any visible signs of phase separation or drug precipitation. 

**Figure 2:**
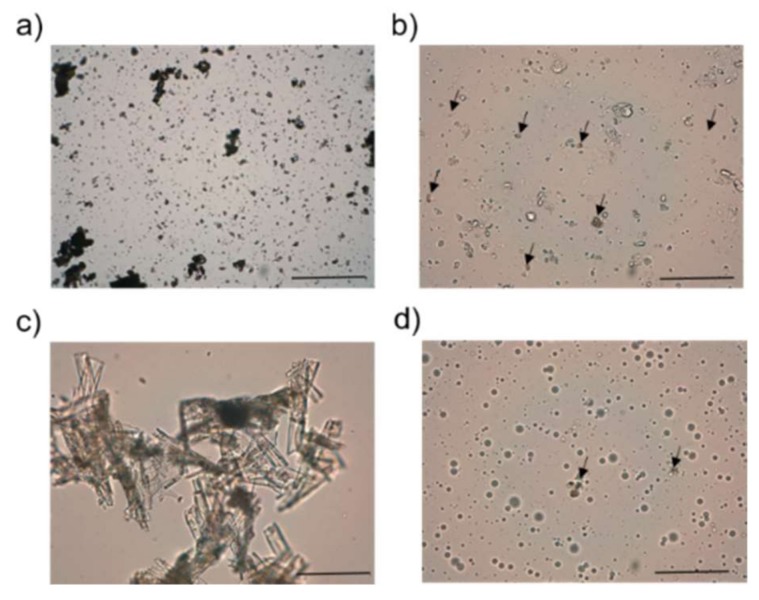
Optical photomicrographs at 40x magnification of (**a**) micronized DAP in water, (**b**) O/W cream containing DAP and diluted 1:10, (**c**) DAP in diethylene glycol monoethyl ether (DEGEE)/water (1:10 *v*/*v*), and (**d**) DAP–PLE at a 1:10 dilution. Scale bar = 500 µm. Arrows represent DAP.

Regarding microscope images, plain (data not shown) or DAP (Figure 2b) cream showed aggregation of the inner oily vesicles, whose sizes ranged from 20 to 60 µm. The particles of micronized DAP (light brown color) dispersed in the O/W cream had sizes ranged from 10 to 60 µm, similar to what was observed in water (Figure 2a). On the other hand, DAP, solubilized in DEGEE, was easily precipitated when water was added (Figure 2c) in the form of big and long crystals (between 100 and 350 µm, approximately). Although no visible signs of drug precipitation were found in DAP–PLE, microphotographs (Figure 2d arrows) revealed some DAP precipitation, consistent with solubilization until saturation level. However, these crystals were smaller than in Figure 2c, with sizes around 40–80 µm. Finally, microscope images revealed PLE as an O/W emulgel. The oily vesicles of MCT with a mean diameter of 35 µm would be stabilized with lecithin and Pluronic F127^®^ gelled aqueous phase. 

Regarding stability studies, DAP cream was shown to be stable during the evaluation time at the three different storage temperatures. Only pH values seemed to increase slightly compared to the initial formulations (Table S1). However, DAP–PLE was not stable over time. Although at 4 °C all parameters, with the exception of noticeable drug precipitation (only 15 days), were maintained for 60 days, at 25 and 40 °C, there was a clear degradation over time. This lack of stability was observed not only in color, pH, and spreadability, but also in organoleptic properties, phase separation, and drug precipitation. After 60 days at 25 and 40 °C, pH decreased (from pH 4.84 to 3.67 and 3.55, respectively). Besides, spreadability increased 1.8-fold after 25 °C storage, probably due to exhudation phenomenon. Moreover, none of the formulations of the DAP–PLE overcame the gravitational stability test (Table S2).

### 3.3. In Vitro Permeation and Penetration Studies in Pig Ear Skin

**Figure 3:**
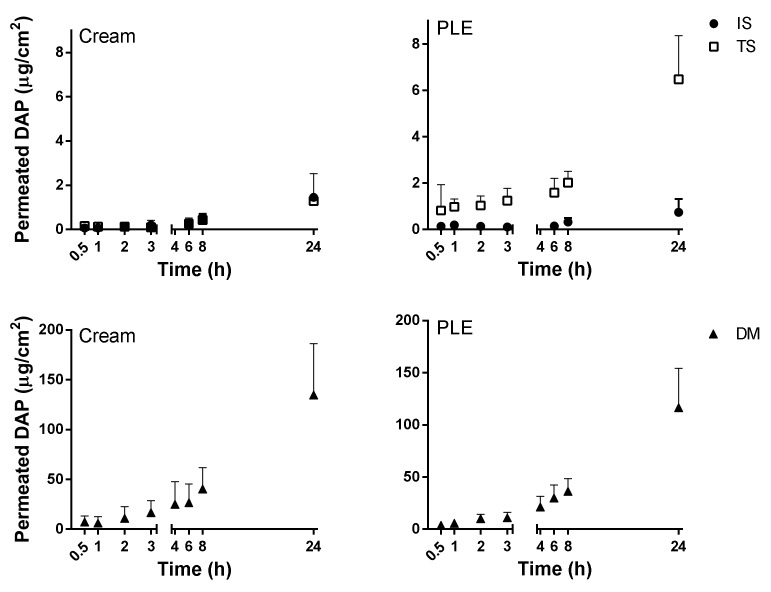
Amount of DAP permeated in vitro for the cream and PLE in the three different skin models evaluated using pig ear skin after 24 h of application. Abbreviations: IS, intact skin, TS, tape stripped; DM, dermal membranes.

Skin CL lesions can vary widely across the infecting *Leishmania* strains, from small dry lesions to large ulcers or even keratolytic nodules. As an attempt to mimic the conditions of skin lesions observed in CL, the permeation studies were conducted in three skin models: IS, TS (removal of the SC, which was used to mimic the damaged skin), and DM (skin without epidermis). DM has been considered as a good model for ulcerated skin lesions [18,21,22]. The results are summarized in Table 5 and Figure 3. As was expected, it was determined that there were higher cumulative amounts of DAP in the receptor compartment 24 h after dosing in DM than in TS (around 20-times higher) and IS (around 100-times higher) for both DAP cream and PLE. Regarding the flux, similar and low *J*ss were obtained for PLE and cream in IS (0.05 vs. 0.06 µg/cm^2^/h). These values increased with the use of TS only after application of PLE (0.32 µg/cm^2^/h). Finally, they greatly increased in DM and similarly increased for both DAP cream and DAP–PLE (3.96 vs. 4.22 µg/cm^2^/h). In relation to lag times, similar results were obtained from the two formulations, with values ranging from 2 to 3 h. 

In addition permeated drugs, the amount of DAP retained in the skin after 24 h was also analyzed (Table 5). The cream formulation presented higher capacity to retain the drug in the skin than the PLE. In IS and TS skin, the amount of DAP deposited was around 2-fold higher for the DAP cream than for the DAP–PLE. However, it was 4-fold higher when DM were used (0.68 and 2.87 µg of DAP/mg skin quantified in DM, for DAP-PLE and DAP cream, respectively). 

### 3.4. Evaluation of Efficacy in L. major-infected BALB/c Mice: Tail and Ear Models

**Figure 4:**
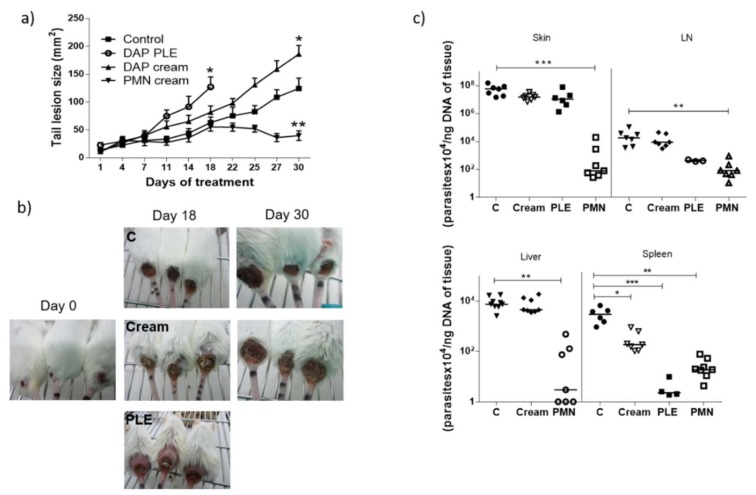
In vivo efficacy study in the tail model. (**a**,**b**) Lesion progression during the treatment with DAP cream or DAP–PLE. PM cream was used as positive control. (**c**) Parasite burden in skin, lymph node (LN), liver, and spleen after 18 days (PLE) or 30 days (cream) of treatment compared with a 30 day treatment of PMN cream. Results are expressed as the median (*n* = 8). Note: * *p* < 0.05, ** *p* < 0.01, and *** *p* < 0.001).

To evaluate the in vivo efficacy of DAP, BALB/c mice were infected with *L. major* metacyclic promastigotes in the base of the tail. Once lesion size increased to 10 mm^2^, topical application of the cream and PLE started. Mice showed a continuous increase in lesion size (Figure 4a,b). In fact, mice treated with DAP cream and DAP–PLE exhibited a faster increment in lesions than non-treated mice. After 18 days of treatment, the topical administration of PLE was stopped because lesion sizes were 2.5-fold higher than in the control group (125 mm^2^ and 50 mm^2^, respectively, *p* < 0.05).

After 30 days DAP cream treatment, lesions had sizes around 186 mm^2^ compared to 125 mm^2^ for control mice (*p* < 0.05) (Figure 4a,b). Furthermore, the amount of DAP quantified in the lesions treated with DAP cream was 9.6 ± 8.5 µg of DAP/mg of skin, which is 0.5 ± 0.4 mg/mice. Finally, parasite burden in skin, lymph node, and liver was similar in mice treated with the cream than in non-treated mice. However, the number of parasites found in the spleen was significantly lower (*p* < 0.05) (Figure 4c). Moreover, when DAP–PLE was administered, parasite burden in lymph node (*p* < 0.05) and spleen (*p* < 0.001) was also significantly reduced. 

**Figure 5:**
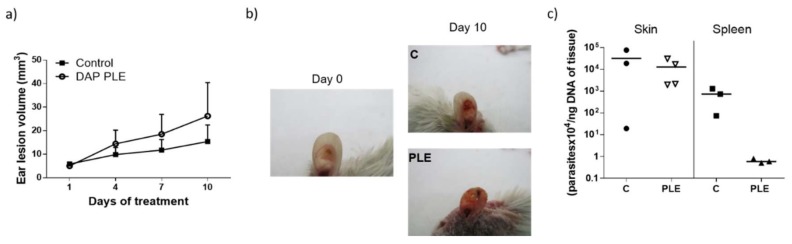
In vivo efficacy study in the ear model. (**a**) Lesion progression during the 10 day treatment with DAP–PLE. (**b**) Skin sections stained with H&E after treatment with PLE. Images are taken at 10x magnification; Scale bar = 300 µm. (**c**) Parasite burden in skin and spleen after 10 days of treatment. Results are expressed as the median (*n* = 5).

On the other hand, in the ear model, treatment with DAP–PLE was started when lesion sizes reached 5 mm^3^ (Figure 5a,b). At day 10, treatment was stopped as the volume in PLE-treated ears was 2.6-fold higher than in the control (24 mm^3^ vs. 9 mm^3^, respectively). Moreover, skin irritation, peeling, and hair loss were found in mice treated with DAP–PLE (Figure 5b). Parasite burden quantified in skin was similar to that in the control. However, parasites quantified in spleen were also reduced, as in the tail model (Figure 5c). 

### 3.5. Plasma Levels, Hematologic Effect, and Iron Content in Spleen after DAP Treatment

**Figure 6:**
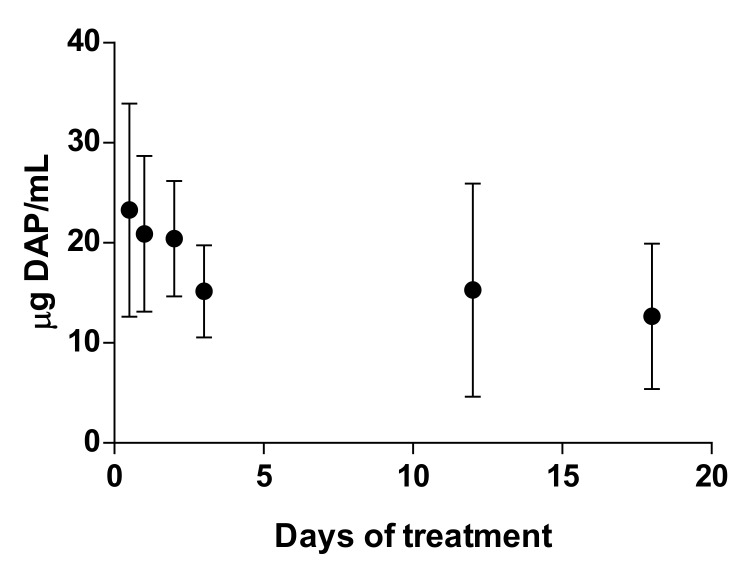
DAP plasma concentration as function of time. Blood was extracted 24 h after the last daily administration. Results are expressed as the mean ± SD (*n* = 6).

Plasma concentrations of DAP were detected 8 h after the first application of DAP cream, with a mean value of 23 µg/mL. After 24 h, this concentration was maintained (21 µg/mL). However, it decreased slightly throughout the days. Finally, 13 µg/mL of the drug was determined at day 18 (Figure 6). Furthermore, as is shown in Table 6, after DAP treatment significantly lower levels of either hemoglobin (12 vs. 17 g/dL, *p* < 0.01) or hematocrit (39 vs. 51%, *p* < 0.01) were detected compared to control untreated mice. Besides, at the end of treatment, a significantly higher amount of iron was quantified in spleen (2.9 vs. 3.5 mg/g, *p* < 0.05). H&E staining of spleen revealed no signs of toxicity after DAP treatment.

## 4. Discussion

DAP has been studied for several dermatological diseases, such as leprosy, dermatitis herpetiformis, or epidermolysis, based on its anti-inflammatory (mostly as inhibitor of neutrophil infiltration) and antimicrobial properties [7]. However, its use after oral administration is widely limited by hematological toxic effects. Topical administration allow its local effects, while avoiding systemic toxicity. At present, a gel formulation of micronized DAP (Aczone^®^) is authorized in some countries for the treatment of acne, with a good security profile [23]. 

Because topical therapy is easy and usually painless, it is an attractive first-line option for the treatment of localized cutaneous leishmaniasis (LCL). In this work, we addressed the preparation of topical formulations for DAP and the assessment of their efficacy in mice with CL lesions by the topical route. 

CL topical therapy represents an enormous challenge, as the target (parasites that harbour macrophages) is localized in the dermis, the deepest skin layer. The accumulation of drugs in this layer is problematic because it has to contend firstly with the SC barrier, and secondly with a rapid clearance to systemic circulation. Moreover, the amount of drug accumulated in the dermis should be higher than a certain threshold of concentration (>IC_50_) and be retained there during the time-to-kill, a parameter that has not been properly considered for antileishmanial agents. It is well-known that drug penetration and permeation into and through the skin is a passive diffusion process, and drug concentration progressively decreases from upper to lower layers [24].

As an advantage in CL lesions, the SC is seriously damaged and this circumstance broadens the optimal physicochemical properties of drugs (MW < 500 Da and LogP 1–4), with the possibility to penetrate into the skin to molecules with MW higher than 500 Da and either more hydrophilic or hydrophobic properties. As a limitation, *Leishmania* spp. infection is associated with local inflammation, increase of blood flow, and skin clearance [25]. On one hand, only interactions with dermal components allow the formation of a reservoir in the dermis [26]. On the other hand, only free drugs are active to exert their action [27]. 

In order to design a formulation of DAP for its topical application, we initially predicted its flux by application of the Potts–Guy equation [28], considering a saturated aqueous solution (0.380 mg/L), and the flux value obtained was 0.102 µg/cm^2^/h. We also determined a value of antileishmanial activity in vitro against *L. major* infected macrophages of 23.2 µg/mL (IC_50_ = 93.7 µM, Table 3). Although the permeability is higher through damaged skin [29], a formulation able to enhance drug penetration is needed. A cut-off value has been established of 1 for the ratio flux/in vitro potency (efficacy index, EI) as a guide element for separating effective and non-effective topical molecules [30,31,32]. This approach is more or less useful for predicting the efficacy of groups of therapeutic agents with targets in the different skin layers, such as antifungal (viable epidermis) or corticosteroid treatments (epidermis and dermis) [33]. According to this criteria, DAP would not be a good topical candidate (EI of 0.0044). However, we decided to test it as we did not observe a correlation between the theoretical EI of some antileishmanial drugs and their topical CL efficacy reported in mice [34,35,36] (see Table 7). Additionally, regarding topical drugs assayed in CL, PM, the only one with efficacy, showed the lowest theoretical flux [6]. On the contrary, sitamaquine (SIT) had a very high flux, although it was not effective [34]. 

In order to enhance DAP permeability, we proposed a pluronic lecithin organogel [37]. We chose this type of formulation because of its ability to enhance the permeability of hydrophilic and hydrophobic drugs, either by its drug solubilization effect or by the interaction of lecithin or the oily phase with the skin, producing its disorganization. Our modus operandi was to use a ratio between the aqueous and oily phase similar to the one currently described in pharmaceutical compounds, with the exception that the most common organic phase, isopropyl palmitate, was replaced by MCT. However, taking into account the composition of the selected formulation (Table 2), we should highlight that it was not an organogel but an emulgel because of the greater amount of aqueous phase compared with the oil content. The vesicles of the oily phase would be stabilized by lecithin and Pluronic F127^®^, as observed in the optical photomicrographs (Figure 2). In fact, the term Pluronic lecithin organogel comprises different types of lecithin-based nanostructured gels, whose molecular organization has not been deeply investigated [37]. 

DAP was solubilized in DEGEE until saturation level (0.5 mg/mL) for its incorporation into PLE in solution and at its maximum thermodynamic activity [38]. According to Fick’s diffusion law that governs the process of penetration of drugs through the skin, only the amount of drug in solution is taken into account in the process. Although drug formulation can increase the amount of the solubilized drug, its flux through the skin is enhanced only if the saturation limit is achieved. Far from the saturation level, the drug could have less tendency to leave the formulation [38,39]. The presence of small particles of DAP would confirm we achieved its saturation level (see Figure 2). On the other hand, DEGEE has been described as a skin penetration modifier. It tends to enhance the permeant skin solubility, resulting in either increased accumulation of the drug in the SC (hydrophobic drugs) or increased permeation [40]. 

Next, we addressed in vitro permeability studies with pig ear skin at infinite dose applications. The PLE was compared with an O/W cream (composition summarized in Table 2), in which DAP was incorporated as a micronized powder at the same final concentration (10% *w*/*v*). This vehicle was the same as that used for the topical administration of PM (used as control for effective treatment) [15]. A priori, a similar and maximal thermodynamic activity of 1 can be assumed for both types of formulations of DAP, either PLE or O/W cream, because of the drug solubilization at its saturation level in each one, although the amount of solubilized drug could be greatly different. The use of suspensions in topical formulations has several advantages, such as sustained release and prolonged levels of drug in the skin layers, while avoiding toxic plasmatic levels of the drugs [41]. However, they may pose problems related to stability and feeling. In detail, Aczone^®^ (the commercialized trademark of DAP) consists of a suspension of the drug in a hydrogel with 25% DEGEE. In this formulation (at 5% *w*/*v* of drug), one-third of the drug is dissolved and two-thirds are suspended as small particles. DAP particles can accumulate in the pilosebaceous unit, which is very suitable for the treatment of acne [40]. 

PLE and DAPs cream showed similar skin permeation profiles, as shown in Figure 3 and summarized in Table 5. Flux was very low in healthy skin—lower than the theoretical value calculated from the Potts–Guy equation from saturated aqueous solution. As expected [29], the permeation of DAP was slightly enhanced when the SC was partially removed by tape stripping. Additionally, it only increased when SC along with the viable epidermis were removed (in DM). The accumulation of the drug in the skin was also higher in DM (see Table 5) than in the other skin conditions and reached the same absolute values as quantified in the receptor compartment. Results indicated that the epidermis was the major barrier for DAP permeation. On the other hand, the free diffusion between dermis and receptor compartments would allow the use of the flux parameter as a predictor of efficacy in DAP topical formulations, because the concentration of the drug in this skin layer is, apparently, the key to its activity [34,42]. The similar and low fluxes (Table 5) obtained from both types of formulations in all skin conditions suggest a possible precipitation of DAP onto the skin upon DGEE absorption from PLE, as previously reported [41].

Although we obtained very low flux values, we decided to evaluate the efficacy of the formulations in BALB/c mice infected with *L. major*, applying a high dose of the drug (500 mg/kg/day). DAP was non-effective and even aggravated the size and appearance of the lesions in the two mice models evaluated (Figure 4a,b, and Figure 5a,b), especially after PLE application. The amount of DAP accumulated in the skin after 30 days of treatment had a mean value of 0.5 ± 04 mg/mice, around 5% of the daily dose (10 mg). Furthermore, the plasma levels of the drug (80 µg/mice, around 1% of the daily dose) were much higher than what has been reported after chronic administration of 150–500 mg/kg/day in healthy mice [43]. This could probably be due to the higher permeability of skin with CL lesions [44], because the area of treatment was similar. We have had difficulty finding information about DAP toxic levels after administration in rodents, as DAP hydroxylamine (DHA), the main metabolite responsible for its side effects, is not produced in mice [45]. Due to this fact, mice are not considered a good model for investigating systemic toxicity of DAP.

Clinical signs of DAP effects in skin (desquamation and hair loss) were more evident after application of PLE, either in the base of the tails or in the ears (see Figure 4b and Figure 5b). We did not evaluate the effect of unloaded PLE, but PLE with IPP as an oil phase had a good safety profile. On the other hand, DEGEE is also considered safe until 40% and DAP–PLE contained a lower percentage (20%). However, the addition of DEGEE to the PLE could produce a higher interaction with the skin and produce irritancy. On the other hand, anemia and associated increase of iron content in the spleen were detected (Table 6). We suggest that the accumulation of iron in the spleen could be responsible for the partial parasite clearance in this organ (*p* < 0.001). The effect played by iron metabolism in the interaction between host and parasite is being investigated as a potential leishmanicidal strategy. Both macrophages and parasites absolutely need iron to survive and their levels must be delicately regulated to support macrophagic killing mechanisms during infection. While some studies showed the importance of iron deprivation in decreasing susceptibility to *Leishmania* infection by limiting the amount of iron available to the parasite, others indicated that it may favour the proliferation of parasites by damaging the immune system [46]. On the other hand, iron-loaded BALB/c mice infected with *L. major* showed reduced parasite burden in organs compared to non-treated mice [47]. Bisti et al. demonstrated that iron not only contributed to diminished infection but also to protecting mice when animals were infected with low doses of parasites.

These *in vivo* results of DAP skin delivery are partially in agreement with the *in vitro* permeation studies performed in IS and TS skin models, in which around 80% of the drug amount that left the formulation remained in the skin and 20% remained in the RC, but not in accordance with the results obtained in DM. Although the skin is seriously damaged when containing CL lesions, these are not really ulcers that expose the dermis, but necrotic and granulomatous tissue that could pose a barrier for permeation of drugs [48]. However, the total amount of DAP that was released from the formulation was higher than that observed in the in vitro experiments (20 µg DAP/mg skin in vivo vs. a maximum of 1 µg/mg skin in ex vivo studies). The permeability of mice skin is generally higher than pig ear or human skin and is markedly increased in CL lesions. Furthermore, the *ex vivo* permeability studies depend only on the process of diffusion, whereas in vivo factors such as blood flow or plasma-binding proteins can accelerate or retard the skin clearance [26]. Blood flow is increased in CL lesions because of associated inflammation [25]. Actually, the efficacy results for PM and inefficacy of buparvaquone (BUR) in CL-infected mice were well correlated with the EI calculated using *ex vivo* experimental flux values obtained with infected mouse skin [44].

Despite systemic toxic levels, it seems that the amount of DAP retained in the skin (20 µg/mg skin) was not enough for parasite clearance, probably due to its accumulation in other skin layers and not in the dermis, according to the *ex vivo* permeability studies for the models of IS or TS. On the other hand, the plasma-binding protein described for DAP was around 70% [49], and as only the free drug is effective, this could be below the threshold of therapeutic efficacy. This estimation also considers that drug detected in the blood was through the dermis (the skin target), which only represented 1% of that total administered drug. There is a great interest in finding any correlation between pharmacokinetic (PK) or pharmacodynamics (PD) parameters and antimicrobial activities, in order to provide a rational dosing regimen to control the development and spread of resistance. Measures of free drug exposure over a 24 h period (fAUC) in relation to the organism minimum inhibitory concentration (MIC) are correlative with the antimicrobial efficacy for most antibiotic classes, but not for all of them. For example, for β-lactams, maintaining a free drug concentration above the MIC of the organism for a portion of the dosing interval has been shown to best predict microbiologic efficacy [50]. These types of studies are lacking in antileishmanial therapy, even after parenteral administration. Therefore, in topical therapy, the PK/PD considerations are complicated by the need for knowledge of drug distribution among different layers of skin and then into the blood.

A previous study optimized a formulation of DAP in nanoemulsions [51]. The best composition achieved a flux value around 100 µg/cm^2^/h through the porcine epidermis. As we observed systemic toxicity upon application of our formulations with much lower flux values, it seems logical to suppose even higher toxicity with formulations with higher permeation. In order to enhance the safety index of DAP after topical application, a formulation able to modulate its clearance from the dermis would be necessary. Formulations able to generate a depot in the SC after their topical application have been reported because of their components (i.e., propilenglycol) and could have a certain penetration at this skin level [52]. In healthy skin, most of the formulation components cannot penetrate the dermis and modify the diffusivity and clearance of drugs from this skin layer. Thus, it is necessary for the drug to achieve rapid penetration of the epidermis and dermis (governed by formulation and drugs properties) and low clearance from the dermis to the blood circulation (sole function of the drug physicochemical properties). However, we should underline that with partial removal of the SC, more formulation ingredients will have the chance to penetrate the dermis, and perhaps affect the accumulation or clearance of the drug from this skin layer.

Overall, our study indicated that topical therapy with DAP at a dose of 500 mg/kg/day administered for 30 days does not cure CL. Moreover, this schedule of administration produced systemic toxicity. Thus, DAP was not a correct choice for the topical therapy of CL, mostly due to poor dermal retention and inadequate safety index. 

## Figures and Tables

**Table 1:** Physico-chemical properties of dapsone (DAP, 4,4-diaminodiphenylsulfone) of interest in topical delivery.

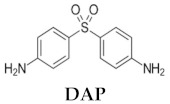	**MW**	**LogP**	**Melting Point (°C)**	**nON**	**nOHNH**
	248.3	0.94	175–176	4	4

Abbreviations: MW, molecular weight; LogP, logarithm of compound partition coefficient between *n*-octanol and water; nON, number of hydrogen bond acceptors; nOHNH, number of hydrogen bond donors.

**Table 2:** Composition of the two blank formulations evaluated in this study.

Phase	Percentage Composition (*w*/*v*)	
	O/W Cream	PLE
Oil Phase	Cetyl alcohol, 3.9%	Medium chain triglycerides (MCT, Miglyol 810^®^), 20.6% Soybean lecithin (Lipoid S100^®^) 4.4%
	Stearic acid, 6%	
	Solid paraffin, 1.5%	
	Liquid paraffin, 7.5% Glyceryl monoestearate, 6.6%	
	White vaseline, 4.5%	
Aqueous phase	Purified water, 70%	Purified water, 58% Pluronic F127^®^, 16%

**Table 3:** *In vitro* activity on *L. major* and *L. braziliensis* promastigotes and amastigotes, and toxicity in mouse peritoneal macrophages, fibroblasts, and keratinocytes after 48 h of treatment with DAP.

EC_50_ (µM)						
*L. major*		*L. braziliensis*		Peritoneal Macrophages	3T3 Fibroblasts	HaCaT Keratinocytes
Promastigotes	Amastigotes	Promastigotes	Amastigotes			
91.8 ± 28.4	93.7 ± 11.4	147.2 ± 42.6	54.5 ± 8.3	1490 ± 300	433 ± 109	3186 ± 208

**Table 4:** Physicochemical characteristics of the cream and PLE formulations (mean ± SD, *n* = 3).

Formulation	Viscosity (Pa.s)	Spreadability (cm)	pH
Cream	2.46 ± 0.61	0.85 ± 0.14	6.52 ± 0.01
DAP cream	2.43 ± 0.55	0.62 ± 0.07	6.57 ± 0.04
PLE	3.31 ± 0.13	0.92 ± 0.17	4.97 ± 0.19
DAP–PLE	2.21 ± 0.04	1.77 ± 0.06	4.84 ± 0.04

**Table 5:** In vitro permeation and penetration values obtained for DAP formulations across pig ear skin after 24 h.

Formulation		*J*ss (μg/cm^2^/h)	*K*p (cm/h)	Lag Time (h)	Cumulative Permeated DAP (µg/cm^2^)	DAP in Skin (μg/mg)
DAP–PLE	IS	0.05 ± 0.02	5.13 × 10^−7^	3	0.73 ± 0.57	0.10 ± 0.07
	TS	0.32 ± 0.16	3.24 × 10^−6^	3	5.22 ± 2.55	0.14 ± 0.03
	DM	3.89 ± 1.71	3.89 × 10^−5^	2	116.47 ± 37.65	0.68 ± 0.54
DAP Cream	IS	0.06 ± 0.01	6.13 × 10^−7^	3	1.29 ± 0.19	0.21 ± 0.02
	TS	0.11± 0.09	1.14 × 10^−6^	3	1.42 ± 1.04	0.28 ± 0.09
	DM	3.96 ± 1.43	3.96 × 10^−5^	2	134.75 ± 51.47	2.87 ± 0.95

Abbreviations: IS, intact skin; TS, tape stripped; DM, dermal membranes; *J*ss, steady-state flux; *K*p, permeability constant.

**Table 6:** Hemoglobin and hematocrit levels after 18 days of DAP cream treatment. Iron accumulation in spleen at the end of treatment (*n* = 8). (* *p* < 0.05 and ** *p* < 0.01).

Group	HGB (g/dL)	HCT (%)	Fe Accumulation in Spleen (mg/g)
Control	17.1 ± 0.5	51.3 ± 1.9	2.89 ± 0.50
Cream	12.4 ± 1.4 **	38.9 ± 5.1 **	3.54 ± 0.50 *

Abbreviations: HGB, hemoglobin; HCT, hematocrit.

**Table 7:** Comparison between the theoretical efficacy index (EI), calculated from the flux (*J*ss) and the EC_50_ value ratio, and the in vivo efficacy in mice for different antileishmanial drugs administered topically.

Drug	*Kp* (cm/h)	*S_sat_* (mg/L)	*J_theor_* (µg/cm^2^/h)	EC_50_	EI	Efficacy	
AmB	1.63 × 10^−8^	0.75	1.23 × 10^−5^	0.1	0.0001	NE	[6]
DAP	2.71 × 10^−4^	0.38	0.103	24.8	0.0044	NE	
SIT	4.96 × 10^−3^	50	247.95	0.93	266.02	NE	[34]
BUR	0.045	0.03	1.35	0.49	2.765	NE	[35]
MIL	2.48 × 10^−4^	0.22	0.05	0.81	0.067	E*	[36]
PM	4.17 × 10^−13^	50	2.08 × 10^−8^	123.4	1.689 × 10^−10^	E	[6]

Abbreviations: AmB, amphotericin B; DAP, dapsone; SIT, sitamaquine; BUR, buparvaquone; MIL, mitelfosine; PM, paromomycin; *Kp*, permeability constant; *S_sat_*, saturated aqueous solution; *J_theor_*, theoretical flux; EI, efficacy index; NE, no efficacy; E, efficacy; E*, there are two studies published: one effective and the other not effective.

## Supplementary Materials

The following are available online at https://www.mdpi.com/1999-4923/11/11/607/s1, Table S1: Stability studies for DAP cream over a period of 60 days at different temperatures. Table S2: Stability studies for DAP PLE over a period of 60 days at different temperatures.
