# Supplementary material for: Evaluation of Skin Permeation and Retention of Topical Dapsone in Murine Cutaneous Leishmaniasis Lesions
Source: Pharmaceutics. 2019 Nov 13;11(11):607. doi: 10.3390/pharmaceutics11110607 (PMC6920985; doi:10.3390/pharmaceutics11110607)
Supplement: Supplementary file 1 [file pharmaceutics-11-00607-s001.pdf]

pharmaceutics

MDPI

Supplementary Materials: Evaluation of Skin Permeation and Retention of Topical Dapsone in Murine Cutaneous Leishmaniasis Lesions

Esther Moreno, Alba Calvo, Juana Schwartz, Iñigo Navarro-Blasco, Elena González-Peñas, Carmen Sanmartín, Juan Manuel Irache and Socorro Espuelas

Table S1. Stability studies for DAP cream over a period of 60 days at different temperatures.

DAP cream	15 days			30 days			45 days			60 days		
	4 °C	25 °C	40 °C	4 °C	25 °C	40 °C	4 °C	25 °C	40 °C	4 °C	25 °C	40 °C
pH	6.97	7.10	6.80	6.99	7.06	6.85	7.05	7.02	6.78	7.08	7.15	6.68
Spreadability (cm)	-			-			-			0.66	0.69	0.72
Colour	Opaque white			Opaque white			Opaque white			Opaque white		
Organoleptic properties	Smooth, high consistency, odourless			Smooth, high consistency, odourless			Smooth, high consistency, odourless			Smooth, high consistency, odourless		
Phase separation	No			No			No			No		
Drug precipitation	-			-			-			-		
Gravitational stability	√			√			√			√		

Table S2**.** Stability studies for DAP PLE over a period of 60 days at different temperatures.

DAP PLE	15 days			30 days			45 days			60 days		
	4 °C	25 °C	40 °C	4 °C	25 °C	40 °C	4 °C	25 °C	40 °C	4 °C	25 °C	40 °C
pH	4.80	4.31	3.74	4.85	3.97	3.78	4.81	3.88	3.46	4.88	3.67	3.55
Spreadability (cm)	-			-			-			1.95	3.27	-
Colour	Beige		Yellow	Beige	Yellow		Beige	Brown		Beige	Brown	
Organoleptic properties	Soft, greasy, fluid, characteristic odour			Soft, greasy, fluid, characteristic odour			Soft, greasy, fluid			Soft, greasy, fluid		
							Characteristic odour	Rancid		Characteristic odour	Rancid	
Phase separation	No			No	Yes		No	Yes		No	Yes	
Drug precipitation	No	Yes		Yes			Yes			Yes		
Gravitational stability	X			X			X			X		
